# Calcineurin inhibitors treatment in kidney transplant recipients is frequently associated with hyperkalemia, acidosis and hypertension but not with increased expression or phosphorylation of the sodium chloride cotransporter

**DOI:** 10.3389/fneph.2026.1917605

**Published:** 2026-10-22

**Authors:** Ludovic Di Ascia, Jennifer Baraka-Vidot, Cyril Ferdynus, Jean-Paul Duong, Céline Bairras Martin, Pierre Merville, Lionel Couzi, Dominique Eladari, Régine Chambrey

**Affiliations:** 1 Université de La Réunion, INSERM, UMR 1188 Diabète athérothrombose Thérapies Réunion Océan Indien (DéTROI), Saint-Pierre, France; 2 Department of Nephrology, Dialysis, and Kidney Transplantation, CHU de la Réunion, Saint-Denis, France; 3 CIC 1410, INSERM, Saint Pierre, France; 4 Université de La Réunion, UMR PIMIT (Processus Infectieux en Milieu Insulaire Tropical), CNRS 9192, INSERM 1187, IRD 249, Saint-Denis, France; 5 Department of medical Informatics, CHU de la Réunion, Saint-Denis, France; 6 Pathology Department, Hôpital Necker, Assistance Publique - Hôpitaux de Paris, Université de Paris, Paris, France; 7 DCRI, Internal Promotion Department, CHU de Bordeaux, Talence, France; 8 Department of Nephrology, Transplantation, Dialysis and Apheresis, CHU de Bordeaux, Bordeaux, France; 9 UMR5164-ImmunoConcEpT, CNRS, Université de Bordeaux, Bordeaux, France; 10 Unité de Médecine de Précision des maladies Métaboliques et Rénales, Service de Néphrologie, CHU Amiens Picardie, Université de Picardie Jules Verne, Amiens, France

**Keywords:** exosomes, familial hyperkalemic hypertension, Gordon syndrome, hypertension, renal acidosis, transplantation

## Abstract

**Background and hypothesis:**

Calcineurin inhibitors (CNIs), widely used after kidney transplantation, may alter signaling pathways regulating the NaCl cotransporter (NCC), potentially causing an acquired form of pseudo-hypoaldosteronism type II (PHAII), characterized by hyperkalemia, metabolic acidosis, and hypertension.

**Methods:**

We assessed the prevalence of a PHAII-like phenotype in kidney transplant recipients (KTRs) receiving CNIs in two independent cohorts: a retrospective cohort (n = 172) and a prospective cohort (n = 58). In the prospective cohort, NCC and phosphorylated NCC (pNCC) abundance were measured in urinary extracellular vesicles (uEVs).

**Results:**

Approximately 25% of KTRs exhibited a PHAII-like phenotype in both cohorts. Contrary to expectations, NCC and pNCC expression in uEVs were lower in CNI-treated KTRs than in healthy volunteers. No differences in NCC or pNCC abundance were observed between KTRs with and without the phenotype. Similarly, kidney transplant biopsies from patients with the phenotype showed NCC and pNCC staining comparable to control biopsies from patients with minimal change disease.

**Conclusion:**

Although hyperkalemia, metabolic acidosis, and hypertension frequently coexist in CNI-treated KTRs, the absence of NCC upregulation does not support the hypothesis that chronic CNI therapy induces the characteristic molecular abnormalities of PHAII.

## Highlights

- What was known CNI therapy in kidney transplant recipients is frequently associated with hypertension, hyperkalemia, and metabolic acidosis—features resembling pseudohypoaldosteronism type II (PHAII). Experimental studies suggested that CNI may induce a PHAII-like state through activation of the NCC pathway.
- This study adds The full PHAII triad is common (~24%) in CNI-treated kidney transplant recipients early after transplantation. However, NCC expression and phosphorylation are decreased—not increased—and are not associated with the PHAII-like phenotype, challenging the hypothesis of NCC overactivation in humans.
- Potential impact These findings question the current paradigm linking CNI toxicity to NCC activation and suggest alternative mechanisms for CNI-induced electrolyte and blood pressure disturbances, with implications for targeted therapeutic strategies.

## Introduction

Since the 80’s, the use of calcineurin inhibitors (CNI) have dramatically improved outcomes in all organ transplants and established kidney transplantation (KT) as the therapy of choice for end stage renal disease (1). CNI include three molecules: cyclosporin A (CsA) and voclosporin, which binds to cytoplasmic cyclophilins (CpN), and tacrolimus (FK506 or Tac), which binds to cytoplasmic Binding-protein 12 (FKBP12) (2). The resulting complexes inhibit calcineurin, a calcium- and calmodulin-dependent serine-threonine phosphatase (3). Calcineurin dephosphorylates, and thereby activates, the transcription factor NFAT (Nuclear Factor of Activated T-lymphocytes). Once activated, NFAT translocates to the nucleus, where it induces IL-2 gene expression and T cell activation (2, 4). Other transcription factors are regulated by calcineurin-dependent dephosphorylation such as forkhead transcription factors (FOXO) and transcription factor EB (TFEB) involved in metabolism, lysosomal biogenesis and autophagy (5).

Calcineurin as well as its ligands CpN and FKBP12 are widely distributed in mammalian tissues and participate to numerous cellular processes and calcium-dependent signal transduction pathways (2, 3, 6). Consequently, CNI use is associated with multiple adverse events (7). Hypertension affects up to 80% of the patients after kidney transplantation (8–10), with reduced incidence in patients with CNI-free regimens (11). Metabolic acidosis is also frequently observed under CNI treatment with ~ 58% of patients affected (12). The reported incidence of hyperkalemia is 5 to 40% among CNI-treated patients (13). Noteworthy, hypertension and acidosis are clinically relevant for physicians in the context of kidney transplantation, because they are associated with higher risk of allograft failure and reduced patient survival (10, 14).

Until recently, the molecular mechanisms accounting for these adverse effects of CNI have remained elusive. However, the association of hypertension, hyperkalemia and metabolic acidosis in kidney transplant recipients (KTR) is reminiscent of the characteristic triad observed in patients affected by pseudohypoaldosteronism type II (PHAII), a rare autosomal dominant monogenic disease, also known as familial hyperkalemic hypertension or Gordon syndrome (15). PHAII is caused by mutations in genes encoding either kinases from the with-no-lysine kinase (WNK) family (16), or their upstream regulators kelch-like 3 (KLHL3) or cullin 3 (CUL3) (17, 18). All these molecules are linked in a signaling cascade that plays a critical role in the regulation of sodium chloride transport by epithelia like the distal nephron (19, 20). In PHAII, hypertension and hyperkalemia result from inappropriate stimulation of electroneutral NaCl absorption by the NaCl cotransporter NCC in the distal convoluted tubule (DCT), which in turn reduces electrogenic Na^+^/K^+^ exchange mediated by principal cells in the renal collecting system (*i.e.*, the connecting tubule and the collecting duct) (21, 22). Metabolic acidosis has also been attributed to reduced distal sodium delivery, which limits ENaC-dependent Na^+^ reabsorption and diminishes the electrochemical gradient required for H^+^ secretion by α-intercalated cells.

Animal studies show that CNI administration in rodents activates NCC via phosphorylation and disrupts the WNK/KLHL3/CUL3 pathway, and that might cause PHAII-like syndrome (23–26). However, CNI toxicity varies considerably among individuals, largely due to genetic polymorphisms affecting CYP3A5 and P-glycoprotein (ABCB1) (27). Hence, it is difficult to assume that the effects observed in rodents after CNI administration are directly translatable to human patients. Therefore, it is not clear whether CNI in humans can provoke PHAII-like syndrome as observed in mice. This is an important question to answer because PHAII is exquisitely sensitive to low dose of thiazide diuretics that might therefore be highly beneficial for CNI-treated KTR.

Therefore, we designed a study that aimed: 1) to determine the prevalence of PHAII-like phenotype in CNI-treated KTR; 2) to determine whether total NCC and phosphorylated NCC abundance in urinary uEVs, used as a proxy for NCC expression, and activation in the kidney, is increased in CNI-treated KTR exhibiting a PHAII-like phenotype.

## Materials and methods

The full details of the methods is detailed in S1 file as supporting information.

### Study design and population

Two complementary studies were conducted in calcineurin inhibitor (CNI)-treated kidney transplant recipients (KTR). First, to determine the prevalence of PHAII-like phenotype, a retrospective cross-sectional study included consecutive KTR attending the 3-month post-transplant visit at Bordeaux University Hospital (April 2017–April 2018) (**Supplementary Figure S1**). Since end stage renal disease is a cause of hypertension, metabolic acidosis, and hyperkalemia, patients with eGFR < 15ml/min per 1.73m2 were excluded. Clinical and laboratory data were collected. Blood pressure was measured according to the recommendation of the international society of hypertension guidelines (28). Hypertension was defined by blood pressure ≥ 140/90 mmHg or by the use of antihypertensive therapies. Metabolic acidosis was defined by a blood [HCO_3_^-^] < 22 mM (in the absence of hypocapnia), or by the need of alkali supplementation. Hyperkalemia was defined by plasma [K^+^] > 4.5 mM or by the need of a potassium chelating agent. Metabolic acidosis and hyperkalemia were confirmed through repeated measurements on independent samples at different time points. Cardiovascular disease was defined by the presence of one of the following: history of myocardial infarction, congestive heart failure, peripheral arteriopathy, abdominal aortic aneurysm, or stroke. Expanded criteria donor was defined by age ≥ 60 years, or age 50–59 years plus two of the following: stroke as the cause of death, preexisting hypertension, or terminal serum creatinine greater than 1.5 mg/dl (132 µmol/l). Delayed graft function was defined by the requirement of dialysis within the first week of transplantation. Proteinuria was defined as a urinary protein/creatinine ratio > 50 mg/mmol of creatinine. Second, to evaluate renal NCC expression and phosphorylation, a prospective multicenter study (Major Activation of NCC in Graft using Urine Evaluation M.A.N.G.U.E) was conducted in Bordeaux and La Réunion (April–August 2018) including KTR at 3 months post-transplant receiving CNI since transplantation (**Supplementary Figure S2**). We excluded patients treated with thiazide diuretics or aldosterone antagonists which can interfere with NCC quantification, patients with significant graft artery stenosis, nephrostomy, or acute disease at the time of inclusion. Like for the retrospective study, deidentified clinical and biological data were collected. Urine samples (∼50 ml) were collected between 7 a.m and 9 a.m and immediately stored at -80 °C with protease inhibitors (Pierce Protease inhibitor tablets, ThermoFisher Scientific, Waltham, MA, USA) for subsequent isolation of uEVs. Of 60 KTR, two were excluded because of a problem with exosome isolation.

The control group consisted of 58 sex-matched healthy volunteers (HV) with no treatment and no history of hypertension, kidney or metabolic disorders. Urine sample of HV was collected in the same conditions than for KTR.

### Isolation of uEVs

uEVs were isolated as previously described (29) using a 2-step centrifugation process. Collection, processing and storage were done according to the position paper from the International Society for Extracellular Vesicles (30). More details are given in **Supplementary Methods**.

### Primary antibodies

The following antibodies were used: rabbit anti human NCC (Millipore AB3553); rabbit anti phosphorylated NCC at threonine-53 (pT53NCC) kindly provided by Dr J Loffing (University of Zurich, Switzerland); rabbit monoclonal anti human Tumor Susceptibility Gene 101 or TSG101, an exosome marker (31) (Abcam Ab125011).

### Immunoblot analyses

uEVs samples were first subjected to 7.5% SDS-PAGE electrophoresis using Criterion cell electrophoresis apparatus for midi gels, 26 wells (BioRad, Hercules, USA). After electrophoresis western blot were performed as described in **Supplementary Methods**. To allow comparison of uEVs isolated from subjects with different urine output gel loading of uEVs samples was normalized to creatinine concentration of urine samples as described previously (31) and inter-gel variability was controlled using a pooled standard sample, as described in **Supplementary Methods**.

### Immunofluorescence on human kidney biopsies

Human renal tissues were selected from the archives of the renal pathology unit (Pathology Department, University Hospital Necker-Enfants Malades, Paris). Human paraffin-embedded kidney sections were stained for NCC and pNCC as described in **Supplementary Methods**. Sections were scanned using a Nanozoomer S60 (Hamamatsu Photonics France). Intensity of fluorescence was measured from microscopy images captured with NDPview^®^ using ImageJ software. For each biopsy, all positive tubules were first delimited, then the mean gray intensity values were measured for each delimited area and the mean gray intensity values were averaged.

### Statistical analysis

Quantitative data are presented as median [interquartile range]. Qualitative data are presented as frequency (N) and percentages (%). Bivariate comparisons of quantitative and qualitative data were assessed using Mann-Whitney test and Fisher’s test, respectively. Multivariate analysis we performed using logistic regression with backward selection. All covariates with a *p*-value below 0.20 in bivariate analyses were entered in the model and removed if they did not reach the statistical significance. All analyses were performed using SAS 9.4 (SAS Institute Cary NC).

Protein abundances were expressed as densitometric units. Statistical comparisons were assessed by Kruskal-Wallis tests followed by Dunn’s multiple comparisons tests or Mann-Whitney tests as appropriate. All analyses were performed using Prism 9 software (GraphPad Software Inc, La Jolla, CA, USA). A two-tailed alpha level of 5% was considered as significant.

### Ethics and regulations

The retrospective study complied with French MR-004 regulations and did not require ethics approval. The prospective study was approved by the institutional review board (CPP Ile-de-France X; NCT03503461), and all participants provided written informed consent.

## Results

### Prevalence of PHAII-like syndrome in the retrospective cohort

We first assessed the prevalence of the PHAII triad (i.e., hypertension, metabolic acidosis and hyperkalemia) in a retrospective cohort of KTR. The process of selection of the participants is described in **Supplementary Figure S1** and general characteristics of the study population are presented in **Supplementary Table S1**.

Among the 172 KTR, hypertension, metabolic acidosis, and hyperkalemia were present in 84.3%, 73.8%, and 38.4% of KTR, respectively; 26.2% exhibited the full PHAII triad. Hypertension with acidosis (without hyperkalemia) was observed in 36.6%, whereas isolated abnormalities were uncommon (**Supplementary Figure S2**).

For sensitivity analyses, patients were sorted according to the presence or absence of the full PHAII-like phenotype. Characteristics of both subgroups are shown in **Table 1**. Patients with the PHAII-like phenotype had more severe hypertension and lower bicarbonate levels, and more frequently received antihypertensive therapies. In multivariate analysis, use of β-blockers, ACE inhibitors/ARBs, and diuretics remained associated, while other clinical factors were not.

**Table 1:** Sensitive analyses according to the presence or absence of PHAII Phenotype in the retrospective cohort.

	No PHAII-like phenotype* N=127	PHAII-like phenotype* N=45	
Univariate analyses			p value
General characteristics			
Age, years	58 [43-68]	56 [49-68]	0.88
Gender M/F, N (%)	85/42 (66.9/33.1)	37/8 (82.2/17.7)	0.05
BMI, kg/m²	23.7 [21.0-27.8]	23.7 [21.2-28.5]	0.83
First transplantation, N (%)	109 (85.8)	36 (80.0)	0.36
Living donors, N (%)	28 (22)	8 (17.8)	0.55
Delayed Graft function, N (%)	20 (16.1)	4 (9.5)	0.29
Expanded criteria donors, N (%)	61 (50.8)	21 (50.0)	0.93
Cardiovascular disease, N (%)	27 (21.3)	15 (33.3)	0.11
Hypertension before transplantation, N (%)	93 (73.2)	28 (62.2)	0.16
Diabetes (including NODAT), N (%)	40 (31.5)	11 (24.4)	0.37
Graft rejection, N (%)	5 (3.9)	3 (6.7)	0.43
eGFR, ml/min per 1.73m^2^	45 [30-55]	42 [33-53]	0.67
Presence of proteinuria, N (%)	44 (34.6)	12 (26.7)	0.33
Treatment			
Mycophenolic acid, N (%)	117 (92.1)	39 (86.7)	0.37
Corticosteroids, N (%)	94 (74.0)	31 (68.9)	0.51
TMP-SMX, N (%)	106 (83.5)	35 (77.8)	0.39
PPI use, N (%)	73 (57.5)	21 (46.7)	0.21
Nephropathy, N (%)			
Glomerular	35 (27.6)	12 (26.7)	0.81
Tubulo-interstitial	17 (13.4)	6 (13.3)	
Vascular	11 (8.7)	6 (13.3)	
Diabetes	12 (9.4)	6 (13.3)	
Polycystic kidney disease	26 (20.5)	6 (13.3)	
Others (including unknown)	26 (20.5)	9 (20.0)	
Blood pressure			
Hypertension, N (%)	100 (78.7)	45 (100)	0.0008
SBP, mmHg	133 [126-144]	142 [130-152]	0.005
DBP, mmHg	78 [70-81]	80 [70-88]	0.04
Antihypertensive therapies			
Number of antihypertensive agents	1 [0–2]	2 [1–2]	0.04
Calcium channel blocker, N (%)	50 (39.4)	24 (53.3)	0.1
Beta-blocker, N (%)	50 (39.4)	27 (60.0)	0.02
ACE inhibitors or ARB, N (%)	39 (30.7)	22 (48.9)	0.03
Diuretic except thiazides, N (%)	31 (24.4)	5 (11.1)	0.01
Thiazide, N (%)	7 (5.5)	5 (11.1)	0.3
Others, N (%)	11 (8.7)	7 (15.6)	0.26
Acid-base			
Acidosis, N (%)	82 (64.6)	45 (100)	<0.0001
Blood [HCO_3_^-^], mM	22 [20-24]	19 [18-21]	<0.0001
Blood [HCO_3_^-^] < 22 mmol/L, N (%)	51 (40.2)	37 (82.2)	<0.0001
Alkaline supplementation, N (%)	61 (48.0)	32 (71.1)	0.008
Potassium			
Hyperkalemia, N (%)	21 (16.5)	45 (100)	<0.0001
[K^+^], mM	4.2 [4-4.44]	4.8 [4.6-5]	<0.0001
[K^+^] >4.5 mmol/L, N (%)	20 (15.7)	45 (100)	<0.0001
Potassium chelating agent, N (%)	2 (1.6)	6 (13.3)	0.04
Multivariate analysis	Odds ratio [95% CI]		p value
Beta-blocker	2.1 [1–4.5]		0.04
ACE inhibitors or ARB	2.6 [1.3–5.4]		0.009
Diuretic except thiazides	0.2 [0.1–0.7]		0.01

Quantitative data are presented as median [IQR]. Qualitative data were expressed as frequency (N) and percentages (%). All quantitative variables were tested using the Mann-Whitney test. For qualitative variables, the Chi-square test was used, except for the following variables, for which Fisher’s exact test was used: Mycophenolic acid, Graft rejection before inclusion, Others antihypertensive therapy, potassium chelating agent use, thiazide use. TMP-SMX, trimethoprim/sulfamethoxazole; PPI, proton pump inhibitor; SBP, systolic blood pressure; DBP, diastolic blood pressure; eGFR, estimated glomerular filtration rate.

### Prevalence of PHAII-like phenotype in MANGUE cohort

The process of selection of the participants is described in **Supplementary Figure S3** and general characteristics of the study population are presented in **Supplementary Table S2**. General characteristics of the participants from the MANGUE cohort were similar to those from the retrospective cohort except that they were slightly younger (48 [36-62] vs. 57.5 [47-68] years, p=0.003), exhibited higher diastolic pressure (83 [74-89] vs. 79 [70-84] mmHg, p=0.005), and less proteinuria (9 (15.8%) vs. 56 (32.6%), p=0.01). Controls from the MANGUE cohort consisted of healthy volunteer (HV) that were selected for the absence of hypertension, kidney disease, or metabolic disorders like diabetes or obesity, and none were taking medications. **Supplementary Table S3** shows that HV were slightly younger than the KTR patients (44.5 [34-52] vs. 48 [36-62] years, p=0.03).

Nearly all the patients (57/58, 98.3%) were treated with tacrolimus. Hypertension, metabolic acidosis, and hyperkalemia were detected in 82.8%, 74.1%, and 37.9% patients, respectively. **Supplementary Figure S4** shows that 14 (24.1%) patients exhibited the full PHAII triad. Hypertension with metabolic acidosis but without hyperkalemia were observed in 20 (34.4%) of patients while 15.5%, 10.3% et 0% had isolated hypertension, acidosis or hyperkalemia, respectively. Noteworthy, the prevalence of hypertension, acidosis and hyperkalemia were similar in both cohorts.

KTR were next sorted according to the presence or absence of PHAII-like phenotype. General characteristics of both subgroups are shown in **Table 2**. In unadjusted analyses, patients with PHAII-like phenotype were significantly older. They received a graft from an older donor, had less preemptive transplantation and most importantly, exhibited significantly lower eGFR than KTR without the PHAII triad. Like in the retrospective cohort they had more severe hypertension. In multivariate analysis adjusted with all these variables, only age remained independently associated (OR = 1.9 [95% IC: 1.2-3.0] p=0.0058).

**Table 2:** Sensitive analyses according to the presence or absence of PHAII Phenotype in the MANGUE cohort.

	No PHAII-like phenotype N=44	PHAII-like phenotype N=14	
Univariate analyses			p value
General characteristics			
Age, years	41.5 [34-56]	56.5 [49-70]	0.002
Gender M/F, N (%)	24/20 (54.5/45.5)	11/3 (78.6/21.4)	0.05
BMI, kg/m²	24 [21.9-28.8]	24.9 [21.9-26.5]	0.99
First transplantation, N (%)	36 (81.8)	13 (92.9)	0.43
Living donors, N (%)	5 (11.4)	1 (7.1)	1
Delayed Graft function, N (%)	3 (6.8)	3 (21.4)	0.14
Expanded criteria donors, N (%)	14 (32.6)	8 (57.1)	0.1
Cardiovascular disease, N (%)	6 (13.6)	4 (28.6)	0.23
Hypertension before transplantation, N (%)	32 (72.7)	12 (85.7)	0.48
Diabetes (including NODAT), N (%)	11 (25.0)	3 (21.4)	1
Graft rejection before inclusion, N (%)	2 (4.5)	0 (0)	1
eGFR, ml/min/1,73m2	49 [34-67]	33 [27-42]	0.02
Presence of proteinuria, N (%)	9 (20.9)	0 (0)	0.09
Treatment			
Corticosteroids, N (%)	27 (61.4)	8 (57.1)	0.78
Mycophenolic acid, N (%)	42 (95.5)	11 (78.6)	0.09
TMP-SMX, N (%)	38 (86.4)	9 (64.2)	0.06
PPI use, N (%)	17 (38.6)	10 (71.4)	0.03
Nephropathy, N (%)			
Glomerular	12 (27.3)	5 (35.7)	0.31
Tubulo-interstitial	4 (9.1)	2 (14.3)	
Vascular	3 (6.8)	0 (0)	
Diabetes	3 (6.8)	3 (21.4)	
Polycystic kidney disease	5 (11.4)	2 (14.3)	
Others (including unknown)	17 (38.6)	2 (14.3)	
Blood pressure			
Hypertension, N (%)	34 (77.3)	14 (100)	0.05
SBP, mmHg	134.5 [125-145]	144.5 [134-156]	0.03
DBP, mmHg	83 [75-89]	83.5 [76-92]	0.9
Antihypertensive therapies			
Number of antihypertensive agents	1 [0.5-2]	2 [2-3]	0.002
Calcium channel blocker, N (%)	20 (45.5)	7 (50.0)	0.77
Beta-blocker, N (%)	9 (20.5)	5 (33.7)	0.24
ACE inhibitors or ARB, N (%)	11 (25)	7 (11)	0.08
Diuretic except thiazides, N (%)	3 (6.8)	5 (35.7)	0.006
Others, N (%)	7 (15.9)	4 (28.6)	0.43
Acid base			
Acidosis, N (%)	29 (65.9)	14 (100)	0.01
Blood [HCO_3_^-^], mM	20 [18-21]	21 [19-24]	0.34
Blood [HCO_3_^-^] < 22 mmol/L, N (%)	10 (71,4)	23 (52.3)	0.21
Alkaline supplementation, N (%)	21 (47.7)	8 (57.1)	0.54
Potassium			
Hyperkalemia, N (%)	8 (18.6)	14 (100)	<0.0001
[K^+^], mM	4 [3.8-4.3]	4.92 [4.7-5.2]	<0.0001
[K^+^] >4.5 mmol/L, N (%)	8 (18.6)	14 (100)	<0.0001
Potassium chelating agent use, N (%)	0 (0.0)	2 (14.3)	0.06
Multivariate analysis	Odds ratio [95% CI]		P value
Age (1)	1.9 [1.2-3.0]		0.0058

Quantitative data are presented as median [IQR]. Qualitative data were expressed as frequency (N) and percentages (%). All quantitative variables were tested using the Mann-Whitney test. For qualitative variables, the Chi-square test was used, except for the following variables, for which Fisher’s exact test was used: Nephropathy, first transplantation, hypertension before transplantation, Diabetes (including NODAT), Cardiovascular disease, Living donors, Delayed graft function, Mycophenolic acid, Graft rejection before inclusion, Antihypertensive therapies, potassium chelating agent use, beta-blocker, Calcium channel blocker, Beta-blocker, ACE inhibitors or ARB, diuretic except thiazides, TMP-SMX use, Acidosis. TMP-SMX, trimethoprim/sulfamethoxazole; PPI, proton pump inhibitor; SBP, systolic blood pressure; DBP, diastolic blood pressure; eGFR, estimated glomerular filtration rate. (1) Each ten-year increase.

### Total NCC and phosphorylated NCC excretion in uEVs of kidney transplant recipients and healthy volunteers

Urinary total NCC and phosphorylated NCC (pNCC) protein abundances were assessed by immunoblot analyses performed on uEVs collected from KTR and HV. Since KTR have only one kidney (i.e., half of total renal mass of HV) and that uEVs excretion depends on nephron mass, they may excrete less uEVs than HV (32). Thus, to make sure that changes in NCC or pNCC protein abundance detected between KTR and HV truly reflects changes in protein expression and not only reflect a change in uEVs (*i.e.*, total protein) amount, we systematically determined the protein abundance of TSG101, a constitutively expressed uEV marker (31). **Supplementary Figure S5** shows a representative immunoblots for total NCC, pNCC and TSG101 of samples from KTR and healthy volunteers. **Figure 1** shows the protein abundance of NCC, pNCC, and TSG101. TSG101 protein abundance was twice lower in KTR than in healthy volunteers. Surprisingly, NCC and pNCC protein abundance were not increased but rather markedly decreased in uEVs from KTR when compared to HV, even after normalization to TSG101 abundance. A similar trend was noted for the only Csa-treated patients (NCC/TSG and pNCC/TSG: 0.424 vs. 1.778 and 0.300 vs. 1.315, respectively).

**Figure 1:**
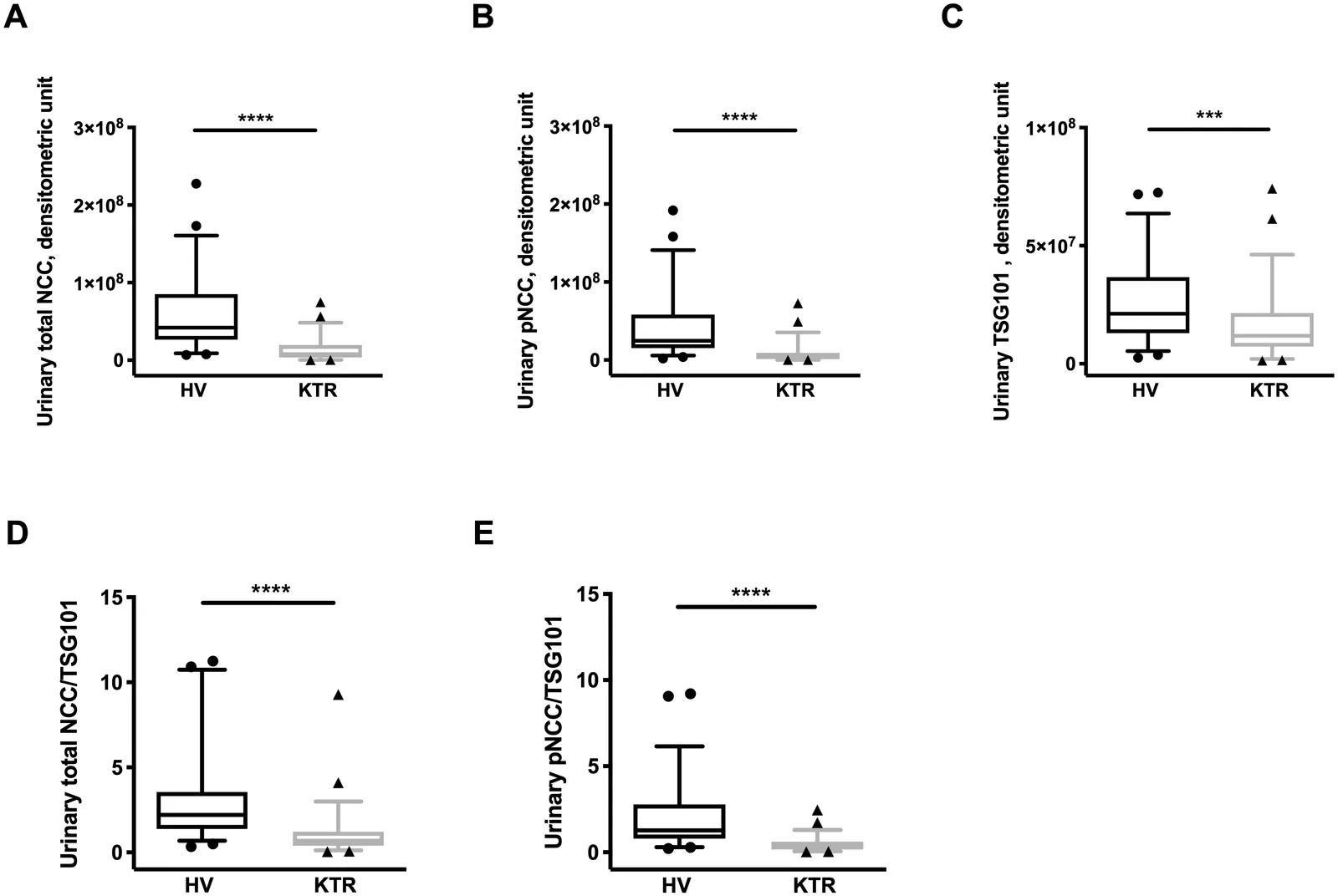
Immunoblot analysis of NCC and pNCC in urinary exosomes of 58 healthy subjects and 58 kidney transplant recipients. Densitometric analyses of immunoblots for NCC and pNCC revealed that both NCC **(A)** and pNCC **(B)** abundance were significantly lower in KTR than in HV. Densitometric analyses of immunoblot for TSG101 **(C)** revealed that TSG101 expression was significantly lower in KTR when compared to HV. Therefore, NCC **(D)** and pNCC **(E)** abundance were normalized to TSG101 abundance. Dataset are presented as box plots with the whiskers representing the 5th and the 95th percentile, N = 58 for each group. Statistical significance was assessed using Mann-Whitney test. ****p < 0.0001 and ***p < 0.001.

The expression of NCC has been reported to be lower in male than in female (32, 33). Therefore, samples from male and female individuals were next analyzed separately. This decrease persisted after stratification by sex (**Figure 2**). Nevertheless, when samples from different sex were analyzed separately, NCC and pNCC abundance were still significantly lower in uEVs from KTR when compared to HV. Kalemia, that may account for sex difference in expression of NCC, was also similar between male and female KTR.

**Figure 2:**
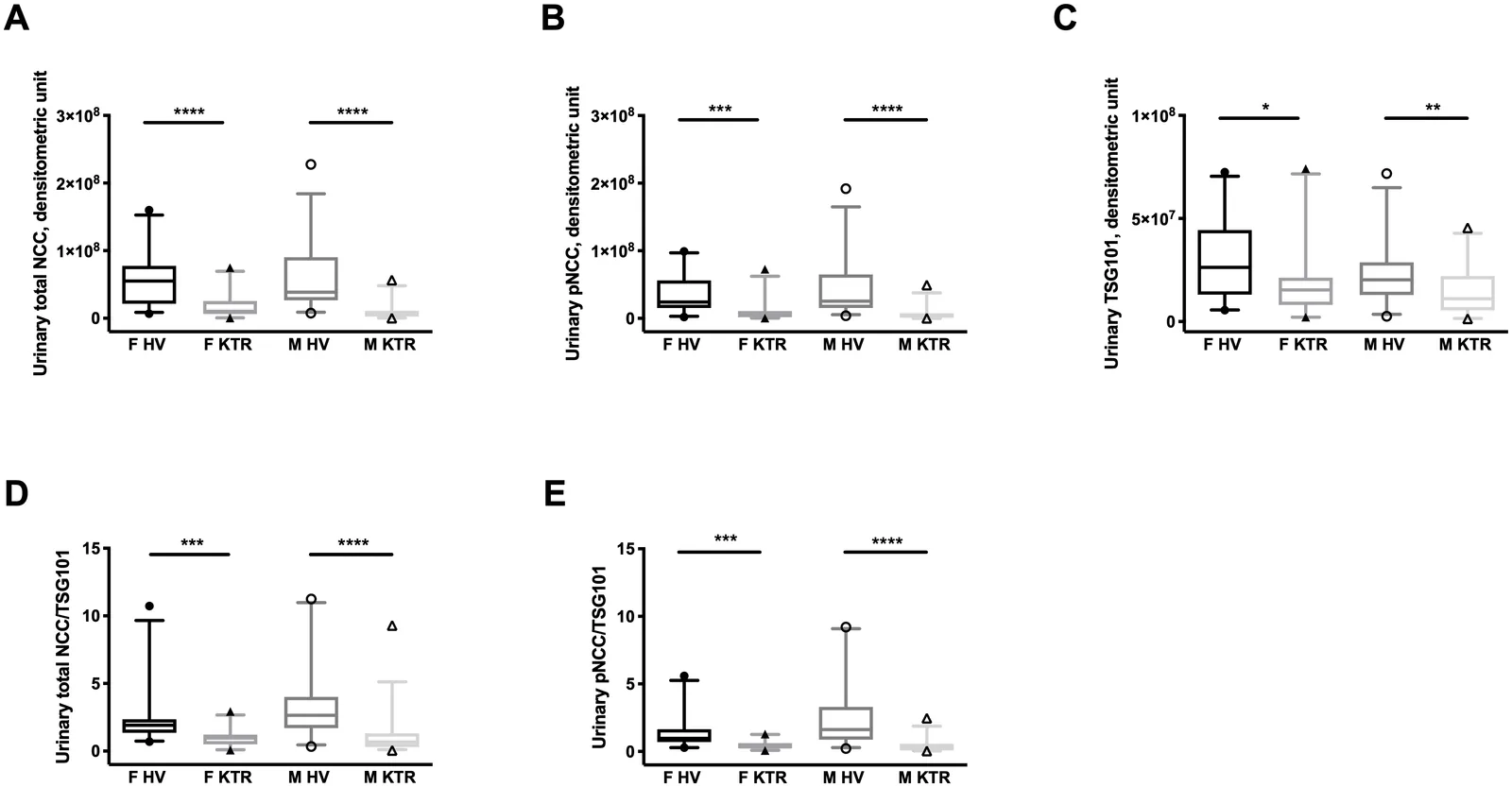
Immunoblot analysis of NCC and pNCC in urinary exosomes of female and male populations of KTR and volunteers. KTR and HV were divided into male (M) and female (F). Both NCC **(A)** and pNCC **(B)** abundances were significantly lower in KTR when compared to HV in both male and female populations. Densitometric analyses of immunoblot for TSG101 **(C)** revealed that TSG101expression in KTR was significantly lower when compared to HV in both male and female populations. Therefore, NCC **(D)** and pNCC **(E)** abundances were normalized to TSG101 abundance. Dataset are presented as box plots with whiskers representing the 5th and the 95th percentile, N = 23 females for both KTR and HV. N = 35 males for both KTR and HV. Statistical significance was assessed using Kruskal-Wallis test. ****p < 0.000, ***p<0.001, **p<0.01 and *p<0.05.

Data obtained from humans can exhibit much more variability than those obtained in laboratory animals. Thus, we next hypothesized that CNI can induce a PHAII-like syndrome only in some patients. We next compared NCC and pNCC protein abundance in KTR with the PHAII triad vs. KTR without the PHAII triad. However, as shown in **Figure 3**, no differences in NCC or pNCC abundance were observed between KTR with or without the PHAII-like phenotype. When then performed a stratified analysis considering the PHA2 phenotype using a stricter definitions of hyperkalemia (serum potassium > 5.0 mmol.L). Again, no significant differences in NCC or pNCC abundance were observed between KTR with or without the PHAII-like phenotype (10.7–10^5^ vs 9.79–10^5^ p=0.72 for NCC, 79.5–10^5^ vs 68.2–10^5^ p=0.95 for pNCC, 0.45 vs 0.48 p=0.54 for NCC/TSG, 0.49 vs 0.37 p=0.59 for pNCC/TSG).

**Figure 3:**
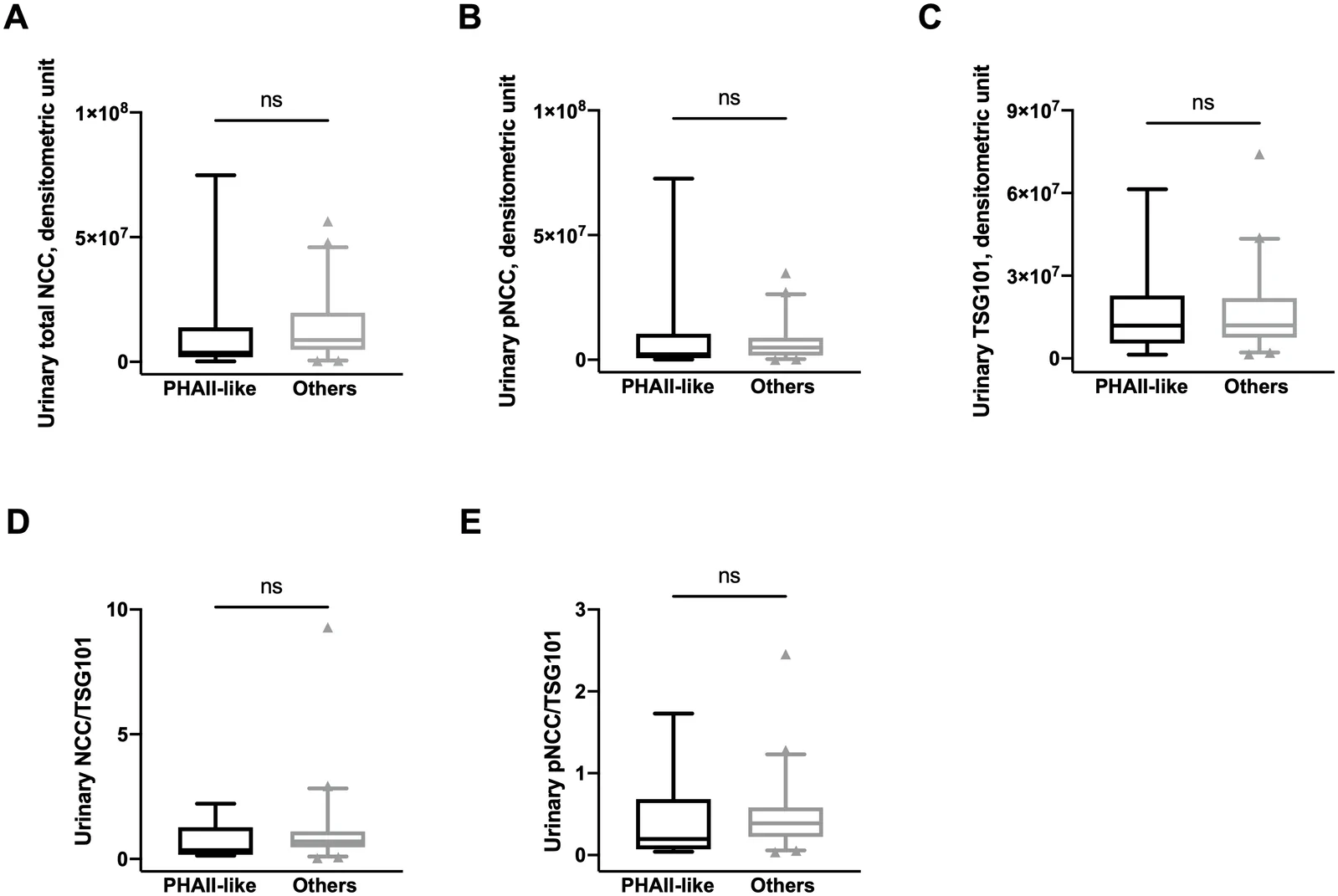
Immunoblot analysis of total NCC and pNCC in urinary exosomes of KTR presenting a PHAII-like phenotype (PHAII-like phenotype) or not (others). Patients from the KTR population were divided into two groups: 1) patients who displayed a combination of hypertension, metabolic acidosis and hyperkalemia (PHAII-like phenotype), and 2) the others. No difference was observed for total NCC **(A)** and pNCC **(B)** abundances between these two groups. Densitometric analyses of immunoblot for TSG101 **(C)** revealed no significant difference between these two groups. Thus, after normalization for TSG101 abundance, total NCC **(D)** and pNCC **(E)** abundances were still not significantly different between the two groups of KTR. Dataset are presented as box plots with whiskers representing the 5th and the 95th percentile, N = 14 KTR exhibiting PHAII-like phenotype and N = 44 KTR without a full PHAII phenotype. Statistical significance was assessed using Kruskal-Wallis test.

Similarly, no difference was detected in NCC and pNCC abundance between KTR with or without hypertension (**Figure 4**). General characteristics of both subgroups are shown in **Table 3**. Same absence of difference was observed when KTR were sorted for the presence of metabolic acidosis or of hyperkalemia (data not shown). Kalemia exhibited a weak negative correlation with the expression of NCC, pNCC, NCC/TSG and pNCC/TSG (data not shown). Furthermore, analysis WB from patients with K >5 (n=7 patients) also shows a decreased expression and phosphorylation of NCC indicating that these patients are not different from those that we describe with a upper limit of 4,5(p=0.0029 for NCC, p=0.0125 for pNCC, p=0.0089 for NCC/TSG and p=0.0306 for pNCC/TSG).

**Figure 4:**
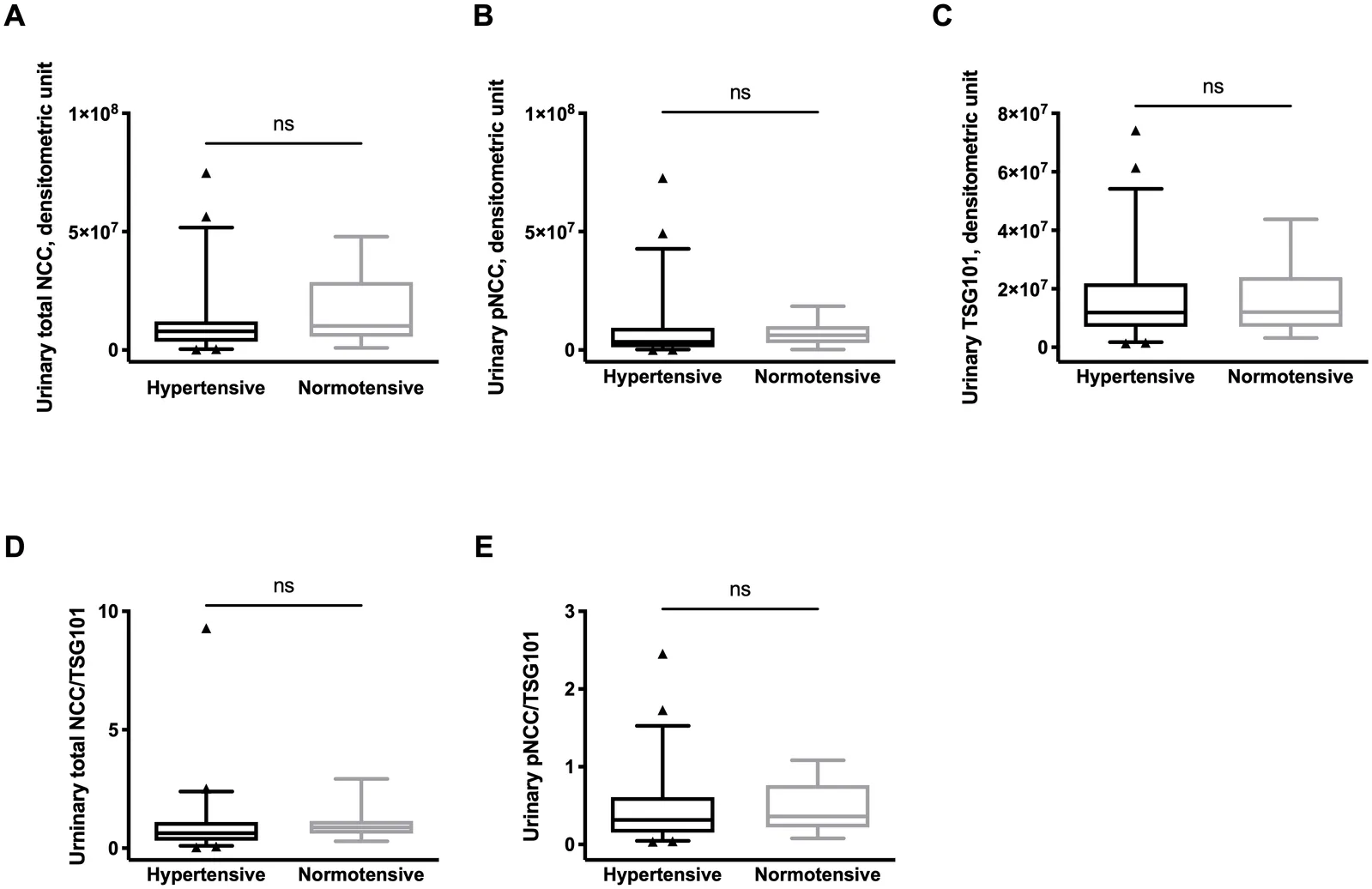
Immunoblot analysis of NCC and pNCC in urinary exosomes of hypertensive and normotensive KTR. Patients from the KTR population were divided into two groups: patients with or without hypertension (Hypertensive and normotensive, respectively). No difference was observed in NCC **(A)** and pNCC **(B)** abundances between the two groups. Densitometric analyses of immunoblot for TSG101 **(C)** revealed no significant difference between the two groups. Thus, after normalization for TSG101 abundance, total NCC **(D)** and pNCC **(E)** abundances were still not significantly different between the two groups of KTR. Dataset are presented as box plots with whiskers representing the 5th and the 95th percentile, N = 48 KTR with hypertension and 10 KTR without hypertension. Statistical significance was assessed using Mann-Whitney test.

**Table 3:** Sensitive analyses according to the presence or absence of Hypertension in the MANGUE cohort.

	No hypertension	Hypertension	
	N=10	N=48	
Univariate analyses			p value
General characteristics			
Age, years	37 (23-41)	49 (39-66)	0.003
Gender M/F, N (%)	3/7 (30.0/70.0)	32/16 (66.7/33.3)	0.04
BMI, kg/m²	24.3 [20.7-27.2]	23.4 [22.4-28.6]	0.48
First transplantation, N (%)	8 (80.0)	41 (85.4)	0.65
Living donors, N (%)	1 (10.0)	5 (10.4)	1
Delayed Graft function, N (%)	1 (10.0)	5 (10.4)	1
Expanded criteria donors, N (%)	2 (20.0)	20 (42.6)	0.29
Cardiovascular disease, N (%)	0	10 (20.8)	0.18
Hypertension before transplantation, N (%)	6 (60.0)	38 (79.2)	0.23
Diabetes (including NODAT), N (%)	4 (40.0)	10 (20.8)	0.2
Graft rejection before inclusion, N (%)	1 (10.0)	1 (2.1)	0.32
eGFR, ml/min/ 1,73m2	47.5 [32 - 81]	42.5 [31 - 59]	0.42
Presence of proteinuria, N (%)	0	9 (19.9)	0.34
Treatment			
Corticosteroids, N (%)	8 (80.0)	27 (56.3)	0.29
Mycophenolic acid, N (%)	10 (100)	43 (89.6)	0.58
TMP-SMX, N (%)	10 (100)	37 (77.1)	0.18
PPI use, N (%)	4 (40.0)	23 (47.9)	0.74
Nephropathy, N (%)			
Glomerular	1 (10)	16 (33.3)	0.06
Tubulo-interstitial	0	6 (12.5)	
Vascular	0	3 (6.3)	
Diabetes	2(20)	4 (8.3)	
Polycystic kidney disease	0	7 (14.6)	
Others (including unknown)	7 (70)	12(25)	
Blood pressure			
Hypertension, N (%)	0	48 (100)	
SBP, mmHg	125.5 [124-127]	140 [129-146]	0.005
DBP, mmHg	75.5 [72-81]	83 [75.5-91.5]	0.08
Antihypertensive therapies			
Number of antihypertensive agents	0	1 (1-2)	<0.0001
Calcium channel blocker, N (%)	0	27 (56.3)	0.001
Beta-blocker, N (%)	0	14 (29.2)	0.1
ACE inhibitors or ARB, N (%)	0	18 (37.5)	0.02
Diuretic except thiazides, N (%)	0	8 (16.7)	0.33
Others, N (%)	0	11 (22.9)	0.18
Acid base			
Acidosis, N (%)	9 (90)	34 (70.8)	0.43
Blood [HCO_3_^-^], mM	19.5 [18 - 20]	21.5 [20-24]	0.01
Blood [HCO_3_^-^] < 22 mmol/L, N (%)	9 (90)	24 (50)	0.03
Alkaline supplementation, N (%)	6 (60)	23 (47.9)	0.49
Potassium			
Hyperkalemia, N (%)	3 (30)	19 (40.4)	0.73
[K^+^], mM	4.18 [3.7-4.5]	4.18 [3.9-4.8]	0.29
[K^+^] >4.5 mmol/L, N (%)	3 (30)	19 (40.4)	0.73
potassium chelating agent use, N (%)	0	2 (4.2)	1

Quantitative data are presented as median [IQR]. Qualitative data were expressed as frequency (N) and percentages (%). All quantitative variables were tested using the Mann-Whitney test. For qualitative variables, the Chi-square test was used. TMP-SMX, trimethoprim/sulfamethoxazole; PPI, proton pump inhibitor; SBP, systolic blood pressure; DBP, diastolic blood pressure; eGFR, estimated glomerular filtration rate.

### NCC expression on human kidney biopsies of CNI-treated patients

Human paraffin-embedded renal biopsies sections from 15 CNI-treated KTR (5 patients with the full PHAII triad, 4 patients with hypertension and metabolic acidosis and 6 hypertensive patients without acidosis and hyperkalemia) were stained for pNCC. Since we could not obtain renal biopsies from HV, they were compared to renal biopsies from 4 patients with minimal change disease taken as controls. Immunofluorescence images are shown in **Figure 5A**. Quantification of pNCC immunostaining is shown in **Figure 5B**. Averaged mean gray intensity of pNCC was not different between CNI-treated KTR and CNI-free patients with minimal change disease. To our knowledge, NCC protein abundance in human patients affected by minimal change disease has not been reported. However, in rats and mouse models of nephrotic syndrome NCC abundance has been repeatedly shown to be slightly reduced (34–36). Thus, the results from these experiments are in line with the results of our immunoblot analyses. Taken together, the results of the present study demonstrate that in KTR patients, CNI treatment did not result in upregulation but rather resulted in a marked downregulation of NCC expression and activation.

**Figure 5:**
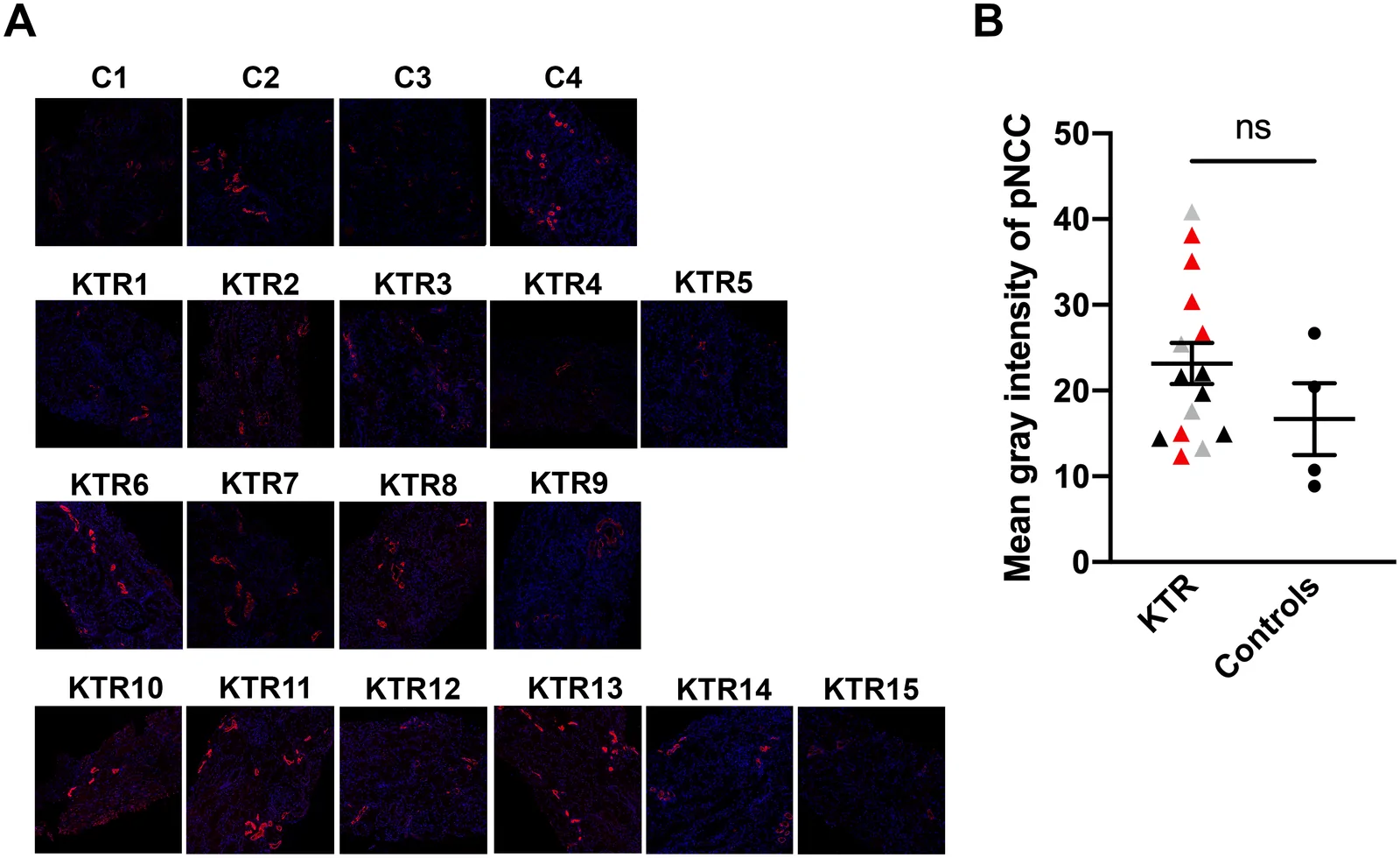
Immunostaining for pNCC of renal biopsies from CNI-treated KTR and control patients. **(A)**. Immunofluorescence images of renal biopsies showing pNCC in 15 CNI-treated KTR [5 patients with the full PHAII triad (KTR1-5), 4 patients with hypertension and metabolic acidosis (KTR6-9), and 6 hypertensive patients without acidosis and hyperkalemia (KTR9-15)] and in 4 patients with minimal change disease (MCD) used as “Normal” controls (C1-4). **(B)**. Measurement of fluorescence level revealed no significant difference in pNCC labeling intensity between CNI-treated KTR and CNI-free patients (with MCD). Scale bars = 100μm. Dataset are presented as mean ± SEM. Statistical significance was assessed using Mann-Whitney test. n.s., Not Significant.

## Discussion

Calcineurin inhibitors (CNI) are widely used immunosuppressive agents but are frequently associated with hypertension and tubular dysfunction, including hyperkalemia and metabolic acidosis. These abnormalities resemble pseudohypoaldosteronism type II (PHAII), a genetic disorder driven by NCC overactivation. Experimental studies have suggested that CNI may induce an acquired PHAII-like state via activation of NCC through the WNK–KLHL3 pathway (23–26, 37). In this study, we show that the PHAII triad (hypertension, acidosis, hyperkalemia) is common in CNI-treated kidney transplant recipients, affecting ~24% of patients at 3 months post-transplant. However, in contrast to the PHAII paradigm, NCC expression and phosphorylation were markedly decreased in KTR compared with healthy controls, and were not different according to the presence of the PHAII-like phenotype or hypertension status. These findings argue against NCC activation as the underlying mechanism.

We did not evaluate directly NCC function, e.g. by testing the effects of thiazide on renal Na^+^ excretion which is a limitation of our study. However, protein expression levels and phosphorylation of NCC have been repeatedly shown in different studies in mice or humans to reflect NCC function (38–40). Therefore, our finding that NCC expression and phosphorylation was decreased in KTR patients with PHA2-like phenotype supports the hypothesis that the phenotype was not caused by CNI-dependent stimulation of NCC.

Even though, hypertension, metabolic acidosis, and hyperkalemia are frequent complications of CNI in solid organ transplant recipients, no previous study has systematically evaluated the coexistence of these abnormalities. Therefore, the prevalence of PHAII-like syndrome in transplant recipient is not known. Here, we report a relatively high prevalence (~24%) of the PHAII triad in two cohorts of CNI-treated KTR (i.e., a total of 230 KTR). Prevalence of arterial hypertension was very high (84%), like what has been reported in the literature (10). Prevalence of metabolic acidosis in previous studies was quite variable, ranging from 11 to 58% of KTR (41). In the present study, we observed a higher prevalence of metabolic acidosis (~74%). We defined metabolic acidosis by the presence of low plasma [HCO_3_^-^] (i.e., [HCO_3_^-^] < 22mM) while most studies generally use a lower threshold to define normal [HCO_3_^-^] value. Hyperkalemia was less frequent (38%) than hypertension and acidosis, and published data are too limited for meaningful comparison (13). Even though KTR patients frequently exhibited PHA2-like phenotype in our study, our finding that NCC expression and phosphorylation were decreased in these patients does not support the possibility that they had a CNI-induced PHA2. Our results rather favor other mechanisms like for example a decline in renal function. This hypothesis is further supported by the observation that multivariable regression analyses identified only the age as an independently associated variable to PHA2 phenotype. Age is closely correlated with eGFR as renal function physiologically declines with age. However, given our sample size, a lack of statistical power to detect an independent effect of eGFR cannot be ruled out.

Previous human studies assessing the effect of CNI on renal sodium transporters have yielded conflicting results. Hoorn et al. (23), reported increased NCC staining in kidney biopsies from CNI-treated KTR than in biopsies from CNI-free KTR (on azathioprine) or healthy native donors. NCC abundance was not measured by a technique more appropriate for semi-quantitation of membrane proteins like immunoblots. In this study, PHAII-like phenotype was defined by the presence of resistant hypertension (requiring two or more antihypertensive agents), hyperkalemia (plasma potassium recurrently > 5.5 mmol/L) or acidosis (serum bicarbonate < 20 mmol/L, or requiring oral bicarbonate supplements). This definition was stricter than ours and only six of our 230 patients would have met those criteria. Thus, it is not possible to rule out that CNI can cause a true PHAII syndrome in a subset of patients, potentially facilitated by individual susceptibility to these agents. We cannot also exclude a functional upregulation of NCC be in the absence of direct measurement of NCC activity in our cohort.

A subsequent study by Esteva-Font et al. (42), however, reported that CsA-treated KTR exhibited higher blood pressure but similar urinary NCC excretion compared with CNI-free patients, and no correlation between NCC abundance and blood pressure. This suggests alternative mechanisms for CNI-induced hypertension. Supporting this view, a recent study by Capolongo et al. reported that NKCC2 expression in uEVs, from patients treated by CsA (but not by tacrolimus) is increased. This observation indicates that increased sodium reabsorption in the thick ascending limb may be involved. It also suggests that CsA or tacrolimus have different effects (43). Tutakhel et al. (44) showed that in CNI-treated KTR the abundance of both total and phosphorylated NCC in uEVs is higher than in CNI-free KTR but similar as in healthy volunteers. However, the number of patients, particularly in the two control groups, included in this study was quite limited (13 CNI-free KTR and 6 healthy volunteers), blood pressure was not significantly higher in CNI-treated KTR than in CNI-free KTR, and the predominant feature of hyperkaliemia in tacrolimus-treated patients unresponsive to thiazide test argue against an association with a PHAII-like phenotype. Furthermore, another study reported that unlike patients affected by PHAII who are characterized by marked sensitivity to thiazides diuretics (45), KTR treated with tacrolimus exhibit exactly same blood pressure response to chlorthalidone than to amlodipine (46). Finally, Rojas-Vega et al. (33) reported higher NCC expression and phosphorylation in hypertensive compared with normotensive male KTR. Noteworthy, hypertensive patients in that study had lower GFR, were older, and more frequently diabetic—three confounding factors that might impact hypertension. In none of these studies, the acid base status was reported. In the studies from Rojas-Vega et al. (33) and Tutakhel et al. (44), patients did not exhibit hyperkalemia. Furthermore, in another study plasma K^+^ concentration was not higher in CsA-treated KTR when compared to CsA-free KTR (42).

Our study has been performed in KTR treated chronically with CNI. In animal studies (23–26, 37), short CNI treatment (few hours to 2 weeks) induced hypertension through activation of NCC and its regulating kinases (WNK and SPAK). By contrast, during chronic CNI treatment (three to four weeks) down-regulation of NCC and KS-WNK1 has been reported (47–49), suggesting a biphasic effect of CNI on NCC, with an initial upregulation of NCC activity (associated with rise in blood pressure), followed by a secondary downregulation phase. Observations coming from a mouse model with inducible deletion of the calcineurin regulatory subunit (CnB1) in the distal convoluted tubule are consistent with this hypothesis (50). This model is particularly informative because it avoids the adverse effects that can results from CNI overdose. Three weeks after CnB1 deletion, mice developed metabolic acidosis but did not develop hypertension and hyperkalemia. NCC mRNA and protein expression, as well as KS-WNK1 and WNK4 mRNA, were markedly decreased whereas NCC phosphorylation and thiazide-induced natriuresis, a proxy of NCC activity, were unchanged. Ten weeks after CnB1 deletion, both abundance and phosphorylation of NCC were significantly decreased in mice with CnB1 deletion (50). Again, these results are consistent with our observation that chronic use of CNI in KTR leads to decreased rather than increased NCC abundance and phosphorylation.

In conclusion, our results show that the characteristic triad of PHAII is frequently observed in KTR treated with CNI. However, they do not support the possibility that in humans chronic CNI at the usual dosage used in renal transplantation provoke the molecular abnormalities accounting for PHAII.

## Data Availability

The original contributions presented in the study are included in the article/**Supplementary Material**, further inquiries can be directed to the corresponding author/s.
