# Supplementary material for: Calcineurin inhibitors treatment in kidney transplant recipients is frequently associated with hyperkalemia, acidosis and hypertension but not with increased expression or phosphorylation of the sodium chloride cotransporter
Source: Front Nephrol. 2026 Oct 22;6:1917605. doi: 10.3389/fneph.2026.1917605 (PMC13735607; doi:10.3389/fneph.2026.1917605)
Supplement: Supplementary file 1 [file SupplementaryFile1.docx]

**Supplementary Figures**

**for**

**“Calcineurin inhibitors use in kidney transplant recipients is often associated with the symptoms of pseudo-hypoaldosteronism type II but not with upregulation of the renal sodium chloride cotransporter”**

**by**

Ludovic Di Ascia, Jennifer Baraka-Vidot, Cyril Ferdynus C, Jean-Paul Duong-Van-Huyen, Céline Bairras Martin, Pierre-Gilles Merville, Lionel Couzi, Dominique Eladari and Régine Chambrey.

**Supplementary Figures**

**Figure S1: Flow chart of the retrospective cohort of kidney transplant recipients**

**Figure S2: Distribution of hypertension, acidosis and hyperkalemia in the retrospective cohort of 172 kidney transplant recipients 3 months after transplantation.** N= number of patients and (%), % of patients

**Figure S3: Flow chart of the prospective kidney transplant recipients cohort (M.A.N.G.U.E study)**

**Figure S4:** **Distribution of hypertension, acidosis and hyperkalemia in the prospective cohort of 58 kidney transplant recipients 3 months after transplantation.** N= number of patients and (%), % of patients

### Figure S5: Representative immunoblots for NCC, phosphorylated NCC (pNCC) and Tumor Susceptibility Gene 101 protein (TSG101) on urinary exosomes from healthy subjects and kidney transplant recipients. Gel loading of urinary exosomes was normalized to creatinine concentration of urine samples. This normalization allows comparison of subjects with different diuresis. 5 µl of standard (S1) was systematically loaded on each gel to allow comparison of densitometric values obtained from samples loaded on different blot membranes. The standard was also loaded according to creatinuria (S2).
