# Supplementary material for: Calcineurin inhibitors treatment in kidney transplant recipients is frequently associated with hyperkalemia, acidosis and hypertension but not with increased expression or phosphorylation of the sodium chloride cotransporter
Source: Front Nephrol. 2026 Oct 22;6:1917605. doi: 10.3389/fneph.2026.1917605 (PMC13735607; doi:10.3389/fneph.2026.1917605)
Supplement: Supplementary file 2 [file SupplementaryFile2.docx]

**Supplementary detailed Methods**

**for**

**“Calcineurin inhibitors treatment is frequently associated with hyperkalemia, acidosis and hypertension bu not with upregulation of the sodium chloride cotransporter in kidney transplant recipients”**

**by**

Ludovic Di Ascia, Jennifer Baraka-Vidot, Cyril Ferdynus C, Jean-Paul Duong-Van-Huyen, Céline Bairras Martin, Pierre-Gilles Merville, Lionel Couzi, Dominique Eladari and Régine Chambrey.

***Isolation of urinary extracellular vesicules***

A 50 mL urine sample of second urine morning urine was collected in urine collection container for each patient and healthy volunteer and store immediately at -80°C with protease inhibitors (Pierce protease inhibitor tablets, ThermoFischer Scientific, Waltham, MA, USA) to prevent degradation of proteins. Urinary extracellular vesicules were isolated using a 2-step centrifugation process. Frozen urine samples were thawed, homogenized and subjected to 13 000 rpm centrifugation for 12 min at 4°C (Eppendorf Centrifuge 5810R, Rotor F34-6-38) to eliminate whole cells and debris. The supernatant was put aside while the pellet was resuspended in 1 ml isolation buffer (10 mM triethanolamine, 250 mM Sucrose, pH 7.6, containing protease inhibitors), incubated on a shaker for 5 min at room temperature and centrifuged at 13000 rpm for 4 min at 4°C (Eppendorf Centrifuge 5424R, Rotor FA-45-24-11). The supernatant was collected and combined with the supernatant obtained from the first centrifugation. 25 ml of the combined supernatants was centrifuged at 52000 rpm (200 000 x g) for 1 hr at 4°C (Beckman ultracentrifuge, Rotor 70 Ti). The pellet was resuspended in the 25 ml remaining supernatant and centrifuged again. Pellets were solubilized in 100 µl of 1X Laemmli buffer. Samples were incubated at room temperature for 30 min and store at −20°C until use.

***Immunoblot analyses of Urinary extracellular vesiculess***

Immunoblotting was performed on nitrocellulose membranes (Protran premium 0.45 µm, Amersham, USA) using Criterion blotter for midi gels (BioRad, Hercules, USA). Membranes were blocked with 5% non-fat dry milk in PBS for 1 hour at room temperature. The membranes were then incubated with the primary rabbit antibody diluted in PBS containing 1% non-fat dry milk overnight at 4°C (anti NCC 1:2000, anti pNCC 1:10000 or anti TSG101 1:1000). After two brief rinces and 5x5 min washes in PBS containing 0.1% tween 20, blots were incubated with HRP conjugated goat anti rabbit IgG (BioRad, Hercules, USA) diluted in PBS containing 5% non-fat dry milk (1:10000) for 2 hours at room temperature. Blots were briefly rinsed twice and then washed 5 x 5 min with PBS containing 0.1% tween 20. Finally, the membranes were washed for 5 min with PBS before enhanced chemiluminescence detection (Supersignal West pico, Thermo Fischer Scientific, Waltham, MA, USA). Chemiluminescence was visualized on an imaging system (Imager 600, GE Healthcare Life science, PA, USA. Total NCC, pT53NCC and the extracellular vesicules marker TSG101 (Tumor Susceptibility Gene 101) were quantified by densitometric analysis of the immununoblot bands using ImageQuant TL software (GE Healthcare Life science, PA, USA). For total NCC and pNCC, both the dimeric and monomeric forms were quantified. For TSG101, the major band observed at 45 kDa was quantified.

The interval between urine collection, uEV isolation, and subsequent immunoblot analysis was maintained within six months.

***Immunofluorescence on human kidney biopsies***

After deparaffinization and rehydration, sections were heated at 96°C in Dako target retrieval solution, pH 9 (Agilent, Santa Clara, CA) for 30 min. Sections were rinced 3 times with Tris-buffer Saline pH 7.6 for 5 min then blocked with antibody diluent with background reducing components (Agilent, Santa Clara, CA) for one hour at room temperature. Blocking buffer was drained from sections which were then incubated overnight at 4°C with the anti-phosphorylated NCC diluted 1:1200, respectively, in antibody diluent with background reducing components (Agilent, Santa Clara, CA). Sections were washed 3 times for 5 min in TBS containing 0.1% Tween 20. Sections were incubated with anti-rabbit Alexa 555 (Invitrogen, Life Technologies, Eugene, OR) diluted 1:1000 in Tris-buffer saline pH 7.6 for 1 hour at room temperature. Sections were washed 3 times for 5 min in TBS containing 0.1% Tween 20. Sections were mounted using vectashield anti-fade mounting medium (Vector Laboratories, Burlingame, CA).
