# Supplementary material for: Calcineurin inhibitors treatment in kidney transplant recipients is frequently associated with hyperkalemia, acidosis and hypertension but not with increased expression or phosphorylation of the sodium chloride cotransporter
Source: Front Nephrol. 2026 Oct 22;6:1917605. doi: 10.3389/fneph.2026.1917605 (PMC13735607; doi:10.3389/fneph.2026.1917605)
Supplement: Supplementary file 3 [file Table1.docx]

**Supplementary Tables**

**for**

**“Calcineurin inhibitors use in kidney transplant recipients is often associated with the symptoms of pseudo-hypoaldosteronism type II but not with upregulation of the renal sodium chloride cotransporter”**

**by**

Ludovic Di Ascia, Jennifer Baraka-Vidot, Cyril Ferdynus C, Jean-Paul Duong-Van-Huyen, Céline Bairras Martin, Pierre-Gilles Merville, Lionel Couzi, Dominique Eladari and Régine Chambrey.

| **Suppl. Table S1: General characteristics of study population from retrospective cohort (n=172)** | |
| --- | --- |
| **General characteristics** |  |
| Age, years | 57.5 [47-68] |
| Gender M/F, N (%) | 122/50 (70.9/29.1) |
| BMI, kg/m² | 23.73 [21.1-27.8] |
| First transplantation, N (%) | 145 (84.3) |
| Living donors, N (%) | 36 (20.9) |
| Delayed Graft function, N (%) | 24 (13.9) |
| Expanded criteria donors, N (%) | 82 (47.7) |
| Cardiovascular disease, N (%) | 42 (24.4) |
| Hypertension before transplantation, N (%) | 121 (70.3) |
| Diabetes (including NODAT), N (%) | 51 (29.6) |
| Graft rejection before inclusion, N (%) | 8 (4.7) |
| eGFR, ml/min/1,73m2 | 45 [30-55] |
| Presence of proteinuria, N (%) | 56 (32.6) |
| **Treatment** |  |
| CNI CsA/Tacrolimus, N (%) | 14/158 (8.1/91.9) |
| Mycophenolic acid, N (%) | 156 (90.7) |
| Corticosteroids, N (%) | 125 (72.7) |
| TMP-SMX use, N (%) | 141 (82) |
| PPI use, N (%) | 94 (54.7) |
| **Nephropathy, N (%)** |  |
| Glomerular | 47 (27.3) |
| Tubulo-interstitial | 23 (13.3) |
| Vascular | 17 (9.9) |
| Diabetes | 18 (10.5) |
| Polycystic kidney disease | 32 (18.6) |
| Others (including unknown) | 35 (20.3) |
| **Blood Pressure** |  |
| Hypertension, N (%) | 145 (84.3) |
| SBP, mmHg | 135 [127-146] |
| DBP, mmHg | 79 [70-84] |
| **Antihypertensive therapies, N (%)** |  |
| Number of antihypertensive agents | 2 [1-2] |
| Calcium channel blocker, N (%) | 74 (43) |
| ACE inhibitors or ARB, N (%) | 61 (35.5) |
| Beta-blocker, N (%) | 77 (44.8) |
| Diuretic except thiazide, N (%) | 34 (19.8) |
| Thiazide, N (%) | 12 (7) |
| Others, N (%) | 18 (10.5) |
| **Acid-base** |  |
| Acidosis, N (%) | 127 (73.8) |
| Blood [HCO_3_^-^], mM | 21 [19-23] |
| Blood [HCO_3_^-^] < 22 mM N (%) | 88 (51.2) |
| Alkaline supplementation, N (%) | 93 (54.1) |
| **Potassium** |  |
| Hyperkalemia, N (%) | 66 (38.4) |
| [K^+^], mM | 4.3 [4.06- 4.70] |
| [K+] >4.5 mM, N (%) | 65 (37.8) |
| Potassium chelating agent, N (%) | 8 (4.7) |
| Quantitative data are presented as median [IQR]. Qualitative data were expressed as frequency | |
| (N) and percentages (%). IQR: Interquartile Range, BMI: Body Mass Index, CNI: Calcineurin | |
| Inhibitors, CsA: Cyclosporin A, TMP-SMX :Trimethoprim/sulfamethoxazole, PPI: Proton Pump | |
| Inhibitors, SBD: Systolic Blood Pressure, DBP :Diastolic Blood Pressure; | |
| eGFR: estimated Glomerular Filtration Rate. |  |

| **Suppl. Table S2: General characteristics of KTR patients from the MANGUE cohort (n=58)** | |
| --- | --- |
| **General characteristics** |  |
| Age, years | 48 [36-62] |
| Gender M/F, N (%) | 35/23 (60.3/39.7) |
| BMI, kg/m² | 24.3 [22.0-28.5] |
| First transplantation, N (%) | 49 (84.5) |
| Living donors, N (%) | 6 (10.3) |
| Delayed Graft function, N (%) | 6 (10.3) |
| Expanded criteria donors, N (%) | 22 (38.6) |
| Cardiovascular disease, N (%) | 10 (17.2) |
| Hypertension before transplantation, N (%) | 44 (75.9) |
| Diabetes (including NODAT), N (%) | 14 (24.1) |
| Graft rejection before inclusion, N (%) | 2 (3.5) |
| eGFR, ml/min/1,73m2 | 44 [31-60] |
| Presence of proteinuria, N (%) | 9 (15.8) |
| **Treatment** |  |
| CNI CsA/Tacrolimus, N (%)  Tacrolimus trough levels, µg.L  CsA trough levels, ng.L | 1/57 (1.7/98.3)  8.7 (7.3-12.3)  156 |
| Mycophenolic acid, N (%) | 53 (91.4) |
| Corticosteroids, N (%) | 35 (60.3) |
| TMP-SMX use, N (%) | 47 (81) |
| PPI use, N (%) | 27 (46.6) |
| **Nephropathy, N (%)** |  |
| Glomerular | 17 (29.3) |
| Tubulo-interstitial | 6 (10.3) |
| Vascular | 3 (5.1) |
| Diabetes | 6 (10.3) |
| Polycystic kidney disease | 7 (12.1) |
| Others (including unknown) | 19 (32.8) |
| **Blood Pressure** |  |
| Hypertension, N (%) | 48 (82.8) |
| SBP, mmHg | 134 [125-145] |
| DBP, mmHg | 83 [74-89] |
| **Antihypertensive therapies, N (%)** |  |
| Number of antihypertensive agents | 1 (1-2) |
| Calcium channel blocker, N (%) | 27 (46.6) |
| ACE inhibitors or ARB, N (%) | 18 (31.03) |
| Beta-blocker, N (%) | 14 (24.1) |
| Diuretic except thiazide, N (%) | 8 (13.8) |
| Others, N (%) | 11 (19) |
| **Acid-base** |  |
| Acidosis, N (%) | 43 (74.1) |
| Blood [HCO_3_^-^], mM | 21 [19-23] |
| Blood [HCO_3_^-^] < 22 mM, N (%) | 33 (56.9) |
| Alkaline supplementation, N (%) | 29 (50) |
| **Potassium** |  |
| Hyperkalemia, N (%) | 22 (38.6) |
| [K^+^], mM | 4.18 [3.9-4.7] |
| [K^+^] >4.5 mM, N (%) | 22 (38.6) |
| Potassium chelating agent use, N (%) | 2 (3.4) |
| Quantitative data are presented as median [IQR]. Qualitative data were expressed as frequency (N) and percentages (%). | |
| IQR: Interquartile Range, BMI: Body Mass Index, CNI: Calcineurin Inhibitors, CsA: Cyclosporin A, | |
| TMP-SMX :Trimethoprim/sulfamethoxazole, PPI: Proton Pump Inhibitors, SBD: Systolic Blood Pressure, | |
| DBP :Diastolic Blood Pressure; eGFR: estimated Glomerular Filtration Rate. | |

| **Suppl. Table 3 General characteristics of healthy volunteers and KTR of the MANGUE Cohort** | | |  |
| --- | --- | --- | --- |
|  | **Healthy volunteers** | **Kidney transplant recipients** |  |
|  | **N= 58** | **N=58** | **P value** |
| **Age, years, median (IQR)** | 44.5 (34-52) | 48 (36-62) | 0.03 |
| Age < 39 years, N (%) | 22 (37.9) | 17 (29.3) |  |
| Age 40-59 years, N (%) | 34 (58.6) | 24 (41.4) |  |
| Age > 60 years, N (%) | 2 (3.5) | 17 (29.3) |  |
| **Sex ratio, M/F, N (%)** | 35/23 (60.3/39.7) | 35/23 (60.3/39.7) | 1 |
| **BMI, kg/m2** | 22.9 (20.9-25.7) | 24.3 (22-28.5) | 0.34 |
